# mRNA Degradation as a Therapeutic Solution for Mucopolysaccharidosis Type IIIC: Use of Antisense Oligonucleotides to Promote Downregulation of Heparan Sulfate Synthesis

**DOI:** 10.3390/ijms26031273

**Published:** 2025-02-01

**Authors:** Juliana Inês Santos, Mariana Gonçalves, Matilde Barbosa Almeida, Hugo Rocha, Ana Joana Duarte, Liliana Matos, Luciana Vaz Moreira, Marisa Encarnação, Paulo Gaspar, Maria João Prata, Maria Francisca Coutinho, Sandra Alves

**Affiliations:** 1 Research and Development Unit, Department of Human Genetics, National Institute of Health Doutor Ricardo Jorge, INSA I.P., Rua Alexandre Herculano, 321, 4000-055 Porto, Portugal; juliana.santos@insa.min-saude.pt (J.I.S.); mariana.goncalves@insa.min-saude.pt (M.G.); matilde.almeida@insa.min-saude.pt (M.B.A.); ana.duarte@insa.min-saude.pt (A.J.D.); liliana.matos@insa.min-saude.pt (L.M.); luciana.moreira@insa.min-saude.pt (L.V.M.); marisa.encarnacao@insa.min-saude.pt (M.E.); francisca.coutinho@insa.min-saude.pt (M.F.C.); 2 Center for the Study of Animal Science, CECA-ICETA, University of Porto, Praça Gomes Teixeira, Apartado 55142, 4051-401 Porto, Portugal; 3 Associate Laboratory for Animal and Veterinary Sciences, AL4AnimalS, Faculty of Veterinary Medicine, University of Lisbon, Avenida da Universidade Técnica, 1300-477 Lisboa, Portugal; 4 Biology Department, Faculty of Sciences, University of Porto, Rua do Campo Alegre, 4169-007 Porto, Portugal; mprata@ipatimup.pt; 5 Centre for the Research and Technology of Agro-Environmental and Biological Sciences, CITAB, Inov4Agro, University of Trás-os-Montes and Alto Douro, 5000-801 Vila Real, Portugal; 6 Department of Medical Sciences, Campus Universitário de Santiago, Edifício da Saúde, Agra do Crasto, 3810-193 Aveiro, Portugal; 7 Newborn Screening, Metabolism and Genetics Unit, Department of Human Genetics, National Institute of Health Doutor Ricardo Jorge, INSA I.P., Rua Alexandre Herculano, 321, 4000-055 Porto, Portugal; hugo.rocha@insa.min-saude.pt (H.R.); paulo.gaspar@insa.min-saude.pt (P.G.); 8 School of Medicine and Biomedical Sciences (ICBAS), University of Porto, Rua de Jorge Viterbo Ferreira 228, 4050-313 Porto, Portugal; 9 Health Research and Innovation Institute, University of Porto, R. Alfredo Allen 208, 4200-135 Porto, Portugal

**Keywords:** Lysosomal Storage Disorders (LSDs), Mucopolysaccharidosis type III (MPS III), genetic substrate reduction therapy (gSRT), RNA therapeutics, antisense oligonucleotides (ASOs), gapmer ASOs

## Abstract

Mucopolysaccharidosis type IIIC is a neurodegenerative lysosomal storage disorder (LSD) characterized by the accumulation of undegraded heparan sulfate (HS) due to the lack of an enzyme responsible for its degradation: acetyl-CoA:α-glucosaminide N-acetyltransferase (HGSNAT). Classical treatments are ineffective. Here, we attempt a new approach in genetic medicine, genetic substrate reduction therapy (gSRT), to counteract this neurological disorder. Briefly, we used synthetic oligonucleotides, particularly gapmer antisense oligonucleotides (ASOs), to target the synthesis of the accumulated compounds at the molecular level, downregulating a specific gene involved in the first step of HS biosynthesis, *XYLT1*. Our goal was to reduce HS production and, consequently, its accumulation. Initially, five gapmer ASOs were designed and their potential to decrease *XYLT1* mRNA levels were tested in patient-derived fibroblasts. Subsequent analyses focused on the two best performing molecules alone. The results showed a high inhibition of the *XYLT1* gene mRNA (around 90%), a decrease in xylosyltransferase I (XT-I) protein levels and a reduction in HS storage 6 and 10 days after transfection (up to 21% and 32%, respectively). Overall, our results are highly promising and may represent the initial step towards the development of a potential therapeutic option not only for MPS IIIC, but virtually for every other MPS III form. Ultimately, the same principle may also apply to other neuropathic MPS.

## 1. Introduction

The therapeutic potential of RNA-based therapies has gained significant attention, especially with the rapid development of messenger RNA (mRNA) vaccines during the COVID-19 pandemic. However, RNA therapeutics have been under development for over 40 years. One of the most promising strategies in this field involves the use of antisense oligonucleotides (ASOs), a novel class of drugs tailored for personalized medicine. ASOs target RNA based on sequence complementarity, and some of the most widely used types are gapmer ASOs, which mediate mRNA cleavage by recruiting RNase H [1,2,3]. Therapeutic ASOs are chemically modified to enhance their properties, increasing their resistance to nucleases and improving target binding affinity. Phosphorothioate (PS) linkages, along with modifications at the 2ʹ position of ribose, such as 2′-O-methoxy-ethyl (2′-MOE), are frequently employed to improve efficacy of therapeutic oligonucleotides. However, modifications other than PS do not activate RNase H. Gapmer ASOs overcome this by using a central PS core that activates RNase H, with “wings” composed of PS and additional modifications that enhance ASOs efficacy [4,5].

Other than their specificity and ability to target otherwise “undruggable” targets, ASOs have proven particularly effective in treating central nervous system (CNS) disorders. Intrathecal (IT) delivery of ASOs in particular has shown broad CNS distribution and therapeutic efficacy, as evidenced by approved treatments for conditions such as spinal muscular atrophy [6,7], amyotrophic lateral sclerosis [8], and Batten disease [9]. Thus, numerous ongoing studies are exploring the use of ASOs for other neurodegenerative disorders through IT administration, including spinocerebellar ataxia types 1, 2 and 3, Huntington’s disease, Alzheimer’s disease, Dravet syndrome, Angelman syndrome, and progressive supranuclear palsy [3,10].

Mucopolysaccharidoses (MPSs) are a subgroup of lysosomal storage disorders (LSDs), comprising 11 disorders across seven subtypes. These disorders are caused by defects in the degradation of glycosaminoglycans (GAGs), namely dermatan, heparan, keratan, and chondroitin sulfates [11,12,13]. Specifically, MPS type III results from a deficiency in the catabolism of heparan sulfate (HS), with MPS III subtypes A, B, C, and D being caused by deficiencies in heparan-N-sulfatase, N-acetyl-α-D-glucosaminidase, acetyl-CoA:α-glucosaminide N-acetyltransferase, and N-acetylglucosamine-6-sulfatase, respectively. Clinically, these subtypes present with severe CNS degeneration and cognitive decline accompanied by mild somatic disease, unlike other MPS types that may or may not present CNS involvement, while also displaying numerous somatic symptoms, some of which extremely severe and debilitating. While enzyme replacement therapies (ERTs) are available for some MPS types, they do not cross the blood–brain barrier (BBB) and are ineffective for MPS III, which remains an unresolved clinical challenge [13,14].

Given the success of ASOs in treating CNS disorders, this study aimed at investigating their potential as a novel genetic substrate reduction therapy (gSRT) for MPS III. The therapeutic rationale is clear: the HS biosynthetic pathway is well characterized, so we propose using ASOs to downregulate a key gene in this pathway to reduce HS production and accumulation.

Interestingly, substrate reduction therapies (SRTs) have already been approved for certain LSDs, such as miglustat and eliglustat tartrate for Gaucher disease, and miglustat for Niemann–Pick disease type C [15]. Chemical and genetic SRTs have also been explored for MPS diseases, some only at pre-clinical levels, while others have actually reached clinical trials [16]. The chemical approach involves the use of small molecules such as genistein, a soy-derived isoflavone, and rhodamine B, both of which act as non-specific inhibitors of GAGs synthesis, and have been investigated in various studies [17,18,19]. For genistein, an open-label study with 22 MPS patients with neurological impairments demonstrated the safety of high-dose genistein [20]. However, a subsequent phase III double-blind, randomized, placebo-controlled trial using high-dose genistein aglycone failed to show significant reductions in cerebrospinal fluid (CSF) HS levels or cognitive improvements, and showed no significant clinical efficacy in MPS III, with only a modest reduction in urinary GAGs [21].

These results emphasize the need for further investigation, and highlight the risks of choosing a drug that does not directly target the intended pathway as a potential therapeutic agent, in this case the GAG biosynthetic cascade, but that instead acts upon a third party, hoping to trigger feedback mechanisms that will ultimately affect the target. Regarding rhodamine B, a study in 2017 using an MPS I mouse model suggested its potential as a promising standalone or adjunct therapy, particularly for skeletal manifestations. Weekly injections of 1 mg/kg rhodamine B for six months in MPS I knockout mice yielded promising results [22]. However, no clinical trials have been conducted to date, likely due to the reported toxic effects of rhodamine B [23]. Also noteworthy is the fact that the mechanism of action through which rhodamine B inhibits GAG synthesis is completely unknown.

The gSRT developed so far have relied on siRNAs. While showing promising in vitro [19,24,25], their clinical translation remains challenging, primarily due to the complexities of siRNA delivery. Effective nanocarriers capable of crossing the BBB are essential for overcoming these challenges [4,5].

In this study, we used gapmer ASOs (2′-MOE-PS) to downregulate the *XYLT1* gene, which encodes xylosyltransferase I (XT-I), a key enzyme in the early stages of GAGs biosynthesis (HS, chondroitin sulfate and dermatan sulfate). By targeting *XYLT1*, we aim to reduce HS, the specific GAG that is accumulated in affected cells and tissues of MPS III patients, potentially slowing disease progression. This approach offers several advantages not only over other potential LSD treatment options, but also over other forms of SRT, including the potential to treat multiple diseases that share the same accumulating substrates, while ensuring remarkable target specificity. Instead of a “one-compound-for-one-disease” model, our gSRT strategy could support a “one-compound-for-several-diseases” approach, reducing treatment costs and expanding the number of patients who would ultimately benefit from it.

Here, we present a proof of concept for our approach in MPS IIIC (MIM#252930), using patient-derived fibroblasts as a preliminary model. We chose MPS IIIC because, unlike other MPS types, it is caused by defects in an enzyme that is quite challenging: acetyl-CoA:α-glucosaminide N-acetyltransferase (HGSNAT; EC:2.3.1.78). Unlike most MPS-related proteins, this enzyme is not a lysosomal hydrolase but a transmembrane protein of the lysosome. Consequently, therapeutic options for this disease are much more limited, as ERT is not a viable option for these patients. Overall, our results demonstrated significant reductions in *XYLT1* mRNA and protein levels in these fibroblasts, along with a notable decrease in HS levels following ASO treatment.

## 2. Results

### 2.1. Evaluation of XYLT1 mRNA Expression: Effect of Designed Gapmers

Five distinct 2′-MOE-PS gapmer oligonucleotides were designed to downregulate the expression of *XYLT1*, a gene involved in the initial step of the HS biosynthesis (Table 1).

The design considered that gapmers have a central stretch of 10 DNA nucleotides flanked on each side by a wings of 5 nucleotides containing 2′-MOE modifications in the sugar ring too. These modifications increase nuclease resistance, protecting the ends of the ASO, avoid or reduce an immune response, and increase target binding affinity.

Additionally, PS bonds were included throughout the gapmer to increase stability and plasma protein binding, thereby improving its pharmacokinetic properties. Gapmers bind to their target RNA and form a stretch of double-stranded RNA–DNA hybrid that is recognized and disrupted via RNase H-mediated cleavage of the RNA strand.

**Figure 1:**
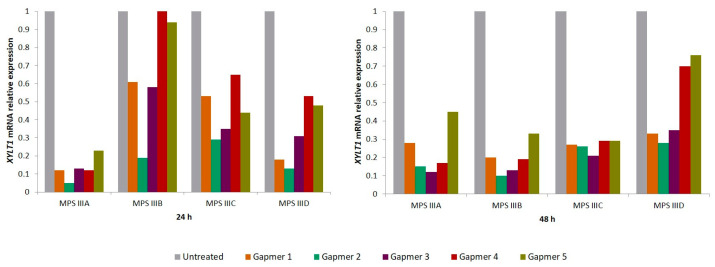
*XYLT1* mRNA relative expression in MPS III (A–D) patients’ fibroblasts, after transfection with five different gapmer antisense oligonucleotides (100 nM). Expression levels were assessed at 24 and 48 h post-transfection. Data represent a single experiment per cell line (*n* = 1).

To test the gapmers’ efficacy in downregulating *XYLT1* mRNA, each gapmer (100 nM) was transfected not only in MPS IIIC patients’ fibroblasts but also into patient-derived cell lines from all the other MPS III substypes: MPS IIIA (MIM#252900), MPS IIIB (MIM#252920) and MPS IIID (MIM#252940). The effect was assessed 24 and 48 h post-transfection, by the relative quantification of *XYLT1* expression, revealing high levels of inhibition. All treated cells showed that *XYLT1* mRNA expression levels reduced at 24 h post-transfection, with most of them displaying a further reduction at 48 h. However, the magnitude of the effect varied between cell lines and among the tested gapmers (Figure 1). Overall, at 24 h, the highest decreases were observed in the MPS IIIA cell line, ranging from nearly 95% to 80%, while the MPS IIIB cell line displayed the smallest reductions, ranging from 80% to only 5%. At 48 h, however, the reductions were more consistent across the four cell lines, varying from a maximum decrease of approximately 90% observed in MPS IIIA and MPS IIIB cells treated with gapmers number 2 and 3 (G2 and G3), respectively. For the MPS IIIC cell line in particular, *XYLT1* levels remained consistently lower at both 24 and 48 h. *GAPDH* was used as an endogenous control for normalization. A similar trend was observed in all cases: the inhibition effect was different between the five gapmers, with G2 and G3 seeming the two most promising ones as they showed a more consistent effect on target expression inhibition at both time points analyzed for the four different cell lines tested. Still, aiming at ensuring the reliability of the results, two novel rounds of experiments were performed in the MPS IIIC cell line (*n* = 3). Overall, the observed results closely mimicked those obtained for the independent cell lines, with G2 and G3 showing a higher knockout power while presenting the lowest standard deviation (see the Supplementary Materials; Figure S1), and thus being selected for a further analysis of their effect on XT-I levels and HS storage.

### 2.2. Inhibition of XYLT1 mRNA with Best-Performing Gapmers: G2 and G3

**Figure 2:**
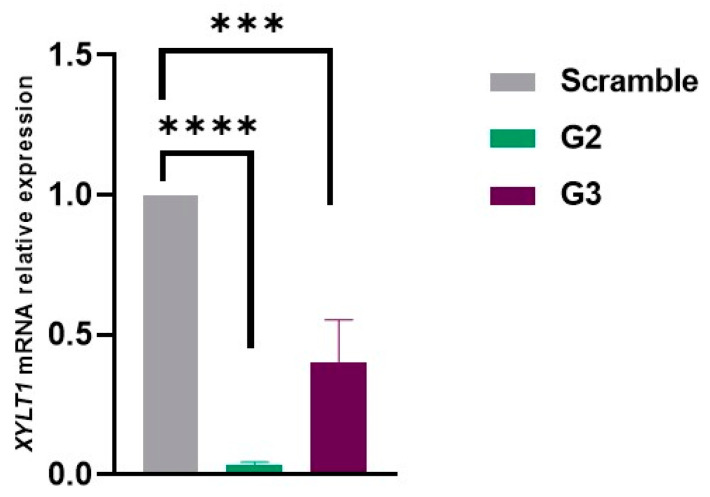
*XYLT1* mRNA relative expression in MPS IIIC patient’s fibroblasts, 24 h after transfection with gapmers G2 and G3, quantified by qRT-PCR in three independent experiments (*n* = 3). *GAPDH* was used as endogenous control. Statistical comparisons between groups were performed using one-way ANOVA and Tukey’s multiple comparisons test as a post hoc comparator (*** *p* < 0.001, **** *p* < 0.0001).

Following the selection of the most promising gapmer ASOs, transfection was repeated on fibroblasts from a MPS IIIC patient, and the effect on RNA levels was analyzed by qRT-PCR 24 h later. For this assay, a scramble gapmer was used as a negative control. Consistent with previous observations, cells transfected with G2 and G3 showed a significant inhibition of *XYLT1* mRNA expression compared to cells treated with the scramble ASO. The inhibitory effect was more pronounced with G2 as expression levels around 10% (corresponding to 90% reduction) were observed. For fibroblasts treated with G3, a decrease of about 70% in *XYLT1* mRNA expression was observed (Figure 2).

### 2.3. Decrease in Xylosyltrasferase I Levels After Treatment with G2 and G3

**Figure 3:**
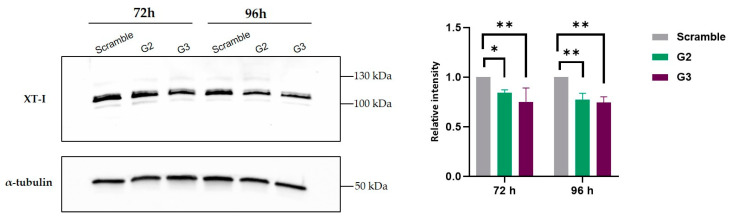
XT-I quantification. The left panel displays a representative Western blot showing XT-I protein levels in MPS IIIC patient’s fibroblasts transfected with gapmers (scramble, G2 and G3), using α-tubulin as the loading control. The right panel displays the quantification of the relative XT-I protein levels, normalized to α-tubulin, from three independent experiments (*n* = 3). Statistical comparisons between G2- or G3-transfected cells and cells transfected with the scramble gapmer were performed using two-way ANOVA followed by Tukey’s multiple comparisons test as a post hoc comparator (* *p* < 0.05, ** *p* < 0.005).

Before we addressed our ultimate goal, which was to check whether a decrease on HS was visible after treatment, we sought to clarify whether the decreases we had seen in *XYLT1* expression levels did actually translate into a decrease in the levels of the protein it encodes, XT-I. XT-I quantification was conducted by comparing band intensities in cells transfected with gapmers G2 and G3 to those transfected with the scramble gapmer. At 72 and 96 h post-transfection, MPS IIIC fibroblasts showed a decrease in XT-I protein levels, as expected. This reduction is in alignment with the observed high inhibition of *XYLT1* mRNA expression, indicating a corresponding decrease at the protein level. α-tubulin was used as control (Figure 3).

### 2.4. Decrease on Heparan Sulfate Levels

Finally, we moved on to quantify our ultimate therapeutic target: HS storage. To achieve this, HS levels were measured by LC-MS/MS in cell pellets collected 6 and 10 days after transfection. Apart from the scramble control, which was also included in all the previous experiments, MPS IIIC fibroblasts transfected with the transfection reagent alone and not exposed to any gapmer, either targeted or scramble, were included in these experiments. This additional condition allowed us to check whether both the scramble gapmer and the transfection itself had any effect on HS accumulation, and is referred to as non-transfected (NT) here.

The HS levels were also measured in a control cell line with no disease, Human Dermal Fibroblasts adult (HDFa), to verify the accumulation pattern. These cells were platted and collected at the same time as the others (6 days). The results were normalized to protein quantity. In MPS IIIC NT fibroblasts, HS levels were 9.92 µg/mg protein. As expected, a very similar amount of HS was detected in fibroblasts transfected with the scramble gapmer: 9.91 µg/mg protein.

The cells treated with gapmers G2 and G3 demonstrated a reduction in HS levels: 6.82 and 8.26 µg HS/mg protein, respectively. For reference, HS levels in HDFa cells had a mean of 3.79 µg HS/mg protein.

When comparing HS levels between HDFa to those of MPS IIIC fibroblasts (NT and/or treated with the scramble ASO), the levels of HS were significantly lower in the first (*p* < 0.0001), as expected.

**Figure 4:**
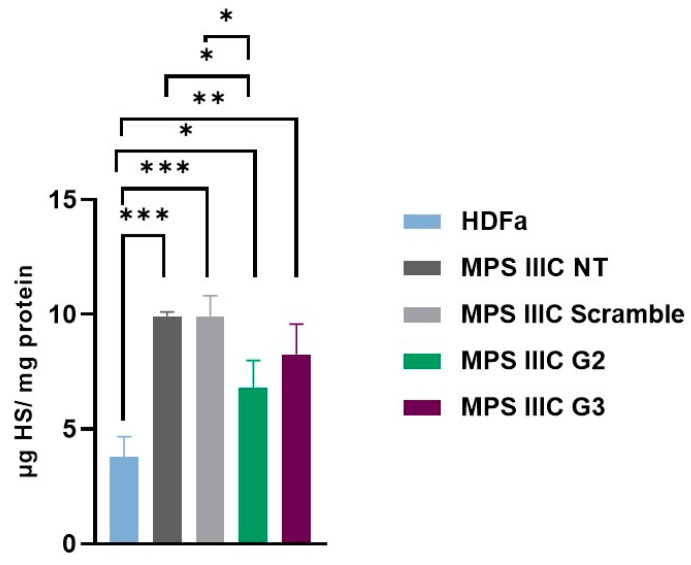
Quantification of heparan sulfate (HS). HS levels were measured in MPS IIIC patient’s fibroblasts 6 days post-transfection with gapmers (scramble, G2 and G3) and in the human dermal fibroblast (HDFa) cell line. Results are expressed in µg HS per mg protein. Statistical comparisons between groups were performed using one-way ANOVA and Tukey’s multiple comparisons test as a post hoc comparator (*** *p* < 0.001, ** *p* < 0.005, * *p* < 0.05). NT: non-transfected MPS IIIC fibroblasts.

The results also showed that the gapmer G2 had a positive impact on HS storage, compared to the scramble gapmer (*p* < 0.05). Although not statistically significant, a decreasing trend is observed when comparing the HS levels of the samples treated with the scramble ASO with the cells transfected with the gapmer G3 (Figure 4).

To check whether the effect of the best performing gapmer, G2, persisted over time, we also tested its ability to reduce HS accumulation in fibroblasts from MPS IIIC patients 10 days after transfection. Gapmer G3 was also included in this analysis, even though the effect seen 6 days after transfection had no statistical significance, in order to verify whether its effect was consistently lower than that of gapmer G2, or needed a longer timeframe to reach statistical significance, unlike gapmer G2.

**Figure 5:**
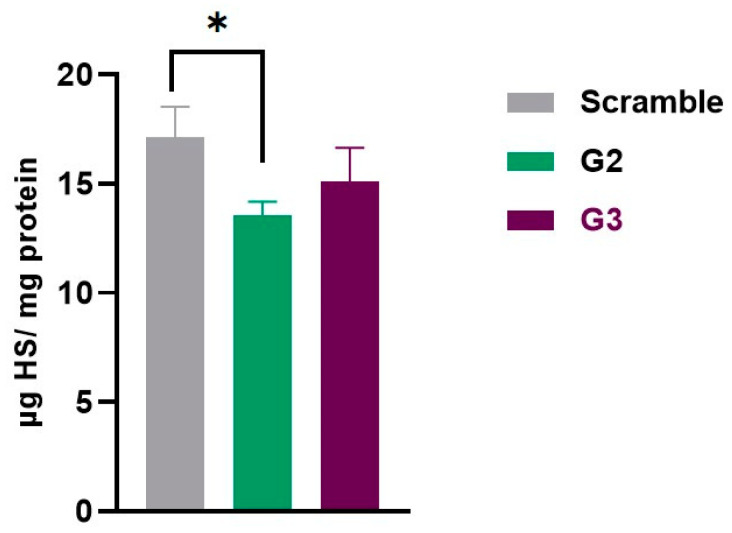
Quantification of heparan sulfate (HS). HS levels were measured in MPS IIIC patient’s fibroblasts 10 days post-transfection with gapmers (scramble, G2 and G3). Results are expressed in µg HS per mg protein. Statistical comparisons between groups were performed using one-way ANOVA and Tukey’s multiple comparisons test as a post hoc comparator (* *p* < 0.05).

The results showed that the effect of gapmer G2 is maintained over time, compared to that of fibroblasts transfected with the gapmer scramble (*p* < 0.05). A trend toward a reduction was detected, once again, with gapmer G3 (Figure 5). Not surprisingly, patient’s fibroblasts showed a pronounced increase in HS storage with time (with means of 9.91 to 17.12 µg HS/mg protein), since these cells are not able to degrade HS.

Overall, from an initial panel of five putative candidates, there was one particular gapmer which fulfilled all criteria to qualify as a potential therapeutic molecule: not only did it reduce its target mRNA and protein levels, but also showed a positive effect in reducing HS storage in patient cells: G2. After a single transfection, it achieved the highest reduction 6 days post-transfection (higher than 20%), but its effect was still statistically significant 4 days later (i.e., 10 days post-transfection).

## 3. Discussion

In this study, we investigated the use of gapmer ASOs as a potential gSRT for MPS IIIC, focusing on downregulating *XYLT1* to reduce the synthesis of XT-I. By targeting XT-I, a key enzyme in GAG synthesis, we aimed to address the pathological accumulation of our target GAG, HS in MPS IIIC. XT-I catalyzes the transfer of UDP-xylose to serine residues on acceptor proteins, a critical step in GAG chain formation. Inhibiting XT-I could broadly reduce GAG synthesis, making it a promising target for multiple MPS subtypes [26]. One of the main reasons for selecting *XYLT1* as the target gene in our initial attempt to downregulate HS synthesis is the compelling evidence from previous studies demonstrating that its inhibition leads to a significant reduction in GAG synthesis, as shown in experiments using siRNAs [27,28].

Mutations in *XYLT1* are implicated in specific genetic disorders, including Desbuquois skeletal dysplasia (DBSD) and Baratela–Scott syndrome. DBSD is an autosomal recessive disease characterized by skeletal dysplasia, joint dislocations, short stature, and facial abnormalities [29,30,31]. Baratela–Scott syndrome presents with short stature, skeletal dysplasia, facial dysmorphisms, and developmental delays [32]. Studies on DBSD have demonstrated that *XYLT1* mutations impair GAG biosynthesis, with Bui et al. reporting low levels of GAGs and proteoglycans in fibroblasts from affected individuals [29]. A recent study by Taieb et al. provided a deeper understanding of the role of *XYLT1* in the onset of the disease, demonstrating that XT-I plays a crucial role in chondrocyte maturation rather than differentiation, highlighting its importance in cartilage development and disease progression [33].

Another important factor supporting the selection of our target gene was the assumption that the transient downregulation of *XYLT1* will not adversely affect cell function, as another enzyme, XT-II, encoded by the *XYLT2* gene, can partially compensate for it. Mutations in *XYLT2* are also associated with a disease phenotype known as spondylo-ocular syndrome, which is characterized by short stature, retinal detachment, amblyopia, nystagmus, heart valve defects, bone fragility, and mild learning difficulties. Again, the levels of GAGs and XT-I activity in the dermal fibroblasts of a patient were lower than in those of healthy subjects [34].

*XYLT1* and *XYLT2* exhibit differential expression across various cell types and tissues. However, both isoforms show broad expression levels in fibroblasts [35] and in the cerebral cortex [36], which is particularly important in the context of our study. Additionally, XT-I and XT-II are expressed in a highly overlapping pattern in adult mice [37]. However, importantly, studies in mouse models and expression analyses across various cell lines have demonstrated that these two xylosyltransferases exhibit both temporally and tissue-specific expression patterns during development [33,35,38,39,40,41]. Additionally, some studies in mouse models have revealed reduced GAG concentrations, along with corresponding alterations in proteoglycan expression, as a result of mutations in both genes [38,40].

Another important argument in favor of our approach comes from a study by Dziedzic et al. in 2012 [28], which, in a similar context of using RNA therapies to downregulate GAG biosynthesis, demonstrated that silencing *XYLT1* alone resulted in a substantial decrease in GAG synthesis, whereas the simultaneous silencing of *XYLT1* and *XYLT2* provided only a marginal additional effect.

Based on these findings, we decided to begin our approach by targeting *XYLT1*, as this strategy could help minimize potential harmful effects for patients, given the possibility that *XYLT2* may partially compensate for the loss of *XYLT1*, a factor that should not be overlooked. However, our objective was to reduce, rather than completely eliminate, *XYLT1* gene expression. It is important to note that ASOs do not achieve complete gene knockout. Instead, their continuous application is expected to result in a controlled reduction in XT-I expression, and, consequently, in HS synthesis, with the aim of halting disease progression.

With that objective, in this study, we used 2′-MOE-PS gapmers, leveraging their enhanced stability, nuclease resistance, and high RNA binding affinity. These second-generation ASOs are known to achieve effective mRNA degradation via RNase H-mediated cleavage without affecting unrelated transcripts, critical for sustained efficacy in vitro and potentially in vivo. This second-generation modification has emerged as the most successful chemistry, having a more favorable safety profile, as suggested by a number of clinical studies. Indeed, the ASOs already available for therapy are 20-mer, 2′-MOE-PS gapmers, such as our tested gapmers, thus showing their potential for clinical translation [42,43]. Initially, we designed and tested five gapmers targeting *XYLT1* and evaluated their effect in four different cell lines. Our rationale for this preliminary assessment was straightforward: although this study focuses on MPS IIIC, any drug or therapeutic molecule acting upon an early step of the GAG biosynthetic cascade has the potential to benefit not only MPS IIIC, but also any other MPS subtypes. Thus, it would be worth assessing its performance in other MPS, particularly those in more urgent need for a therapeutic solution. While only a single experiment was performed for each cell line, the emerging pattern observed across the four MPS III cell lines suggests that G2 and G3 are the most effective, with G2 showing superior results across almost all tested conditions, even in these first assessments. Subsequent experiments in triplicate in fibroblasts from our target disorder alone, MPS IIIC, confirmed these preliminary findings, demonstrating that both gapmers reduced *XYLT1* mRNA and XT-I protein levels, with G2 achieving significant reductions in HS storage in MPS IIIC fibroblasts. It is also worth mentioning that the tested concentration of 100 nM falls within the range typically used for hard-to-transfect cells, such as fibroblasts. It is also within the normal range used for preclinical assessments and later shown to hold sufficient potency to display a therapeutic effect without compromising off-targets.

For instance, Aguti et al. studied the silencing efficiency of gapmer ASOs with 2′-OMe, 2′-MOE, and LNA chemistries. While optimizing allele-specific designs, they used a starting concentration of 100 nM, matching ours. Lower concentrations (10–80 nM for 2′-OMe and 1.25–20 nM for 2′-MOE/LNA) failed to achieve efficient silencing [44].

Therefore, the observed results confirm the efficacy namely of gapmer 2 and highlight its potential for translation to in vivo studies, where it will be possible to better assess the side effects of our therapy. These side effects could be related to the RNA drugs being administered [5] as well as to the pathway we are targeting. Recently, a study by Kleine et al. observed a strong stress response, oxidative stress, senescence and apoptosis following the knockout of *XYLT1* [41], an observation that should not be neglected. Other relevant insights into potential off-targets or cross-effects may also come from the direct measurement of our target’s isoform, XT-II.

Our results validate the proof of concept that 2′-MOE-PS gapmers can effectively downregulate *XYLT1* expression. Furthermore, our data demonstrate that reducing XT-I levels leads to a substantial decrease in HS accumulation, thus acting upon the primary cause of MPS IIIC pathology. This specificity is advantageous for treating MPS IIIC, as HS reduction may alleviate neurological symptoms. The ability of ASOs to cross the BBB via IT delivery enhances their therapeutic potential, overcoming the limitations of traditional SRTs like miglustat and eliglustat tartrate, which fail to target the CNS and often have systemic side effects [15].

For MPS, two chemical compounds, rhodamine B and genistein, have demonstrated the ability to reduce GAG production. However, their mechanisms of action on GAG synthesis remain nonspecific and not fully understood, and their translation to clinical practice has not been successful [19]. In contrast, genetic substrate reduction approaches offer a more targeted strategy, addressing the root cause of the disease with greater specificity, and clinical translation, particularly that using ASOs, appears more feasible. Previous gSRT efforts for MPS relied on siRNAs. Unlike ASOs, siRNAs face significant challenges in delivery, particularly to the CNS, as they require conjugation with other molecules or encapsulation in nanocarriers [3]. For example, Dziedzic et al. carried out the siRNA targeting of *XYLT1*, *XYLT2*, B4GALT7, and B3GALT6 in MPS IIIA fibroblasts, achieving reductions in mRNA and protein levels and impaired GAG synthesis [27]. Similarly, Kaidonis et al. employed shRNAs via lentiviral transfection to inhibit *EXTL2* and *EXTL3* in MPS I and MPS III fibroblasts, yielding comparable results [24]. More recently, Canals et al. tested siRNAs targeting *EXTL2* and *EXTL3* in MPS IIIC fibroblasts, achieving significant HS reductions as early on as in the first 3 days, peaking at 14 days post-treatment. However, translation to the clinic was hampered by the difficulties in the *in vivo* delivery of siRNAs [25].

Our study reinforces that gSRT could be a valuable therapeutic strategy for LSDs with significant neurological involvement. Also noteworthy is that this is the first study to provide a quantitative analysis on the effect one such approach may have upon HS in vitro. In this way, we have assess the true disease biomarker, which is well established as the trigger of the pathophysiological cascade. The only other study that has specifically assessed HS alone is that of Canals et al., carried out in 2015 [25]. However, their analysis was not conducted in a quantitative manner. The results for HS were qualitative, instead, and acquired through immunocytochemistry [25]. 

The successful use of ASOs in CNS-related disorders, such as spinal muscular atrophy [45] and Batten disease [9], further supports their applicability to MPS III [4,5,46,47,48]. With our work, we aim to raise awareness among LSD researchers about the potential of using ASOs as a substrate reduction therapy, and to replicate the promising results obtained with siRNAs, but now utilizing a molecule with more accessible delivery. Future research targeting other genes, such as those already targeted by siRNAs and mentioned earlier, that measure other GAGs and explore additional downstream pathways related to HS accumulation will be important in informing the choice of the best possible target for gSRT, as well as in further validating the specificity of the approach. Equally crucial is the focus on in vivo validation in animal models, such as zebrafish or rodents, to evaluate biodistribution, safety, and long-term efficacy, paving the way for clinical translation.

## 4. Materials and Methods

### 4.1. Gapmer Antisense Oligonucleotides Design

Five different gapmers targeting the *XYLT1* gene, along with one negative control, (scramble ASO) were synthesized by IDT (Integrated DNA Technologies, Coralville, Johnson County, IA, USA). These ASOs are 5-10-5 gapmers that have fully PS-modified linkages with a central segment of 10-mer DNA flanked by 5-mer 2′MOE on both wings.

### 4.2. Cell Culture and Transfection

MPS III (A to D) patient-derived fibroblasts were obtained from the Istituto Giannina Gaslini biobank (Genoa, Italy). A commercial adult Human Dermal Fibroblast, adult (HDFa) cell line available in the lab was used as a control in all experiments. Cells were cultured in Dulbecco’s Modified Eagle Medium (DMEM; Gibco™, Thermo Fisher Scientific, Waltham, MA, USA), high glucose levels, GlutaMAX™ Supplement supplemented with 10% fetal bovine serum (FBS; Gibco™, Thermo Fisher Scientific, Waltham, MA, USA), 1% penicillin/streptomycin (P/S; Gibco™, Thermo Fisher Scientific, Waltham, MA, USA) and 1% Amphotericin B (Gibco™, Thermo Fisher Scientific, Waltham, MA, USA) at 37 °C and 5% CO_2_. The day before ASOs transfection, 1.8 × 10^5^ cells were seeded in a 6-well plate and in 1.125 × 10^6^ in 75 cm^2^ flasks, depending on the subsequent analyses.

Fibroblasts at confluence 65–70% were transfected with the 5 ordered gapmers at 100 nM. For this, each gapmer ASO was mixed with Opti-MEM (Gibco™, Thermo Fisher Scientific, Waltham, MA, USA) containing 10 µL Lipofectamine™ 2000 (Invitrogen™, Thermo Fisher Scientific, Waltham, MA, USA) for each 10 cm^2^ of growth surface area, at room temperature (RT), for 20 min. During this incubation time, the medium of the seeded cells was changed and replaced with the same fresh DMEM referred to above, but without P/S and Amphotericin B. After the referred incubation, gapmer ASOs were added to cells.

Cells were harvested at different time points for different analyses: at 24 h post-transfection for *XYLT1* relative expression analyses; at 72 and 96 h for protein measurement; and at 6 and 10 days for HS quantification. We decided to select different incubation periods to analyze the effects on the mRNA, protein, and HS levels based on the methodology described in previous studies [25,27,28], as well as on the results obtained from our own experience.

### 4.3. Quantitative Real-Time PCR Assay

Total RNA was extracted using GRS Total RNA Kit—Blood & Cultured cells (GRiSP research solutions, Porto, Portugal) following the manufacturer’s protocol. After reverse-transcription with the Ready-To-Go^TM^ You-Prime First-Strand Beads kit (Cytiva, Marlborough, MA, USA), quantitative real-time PCR was conducted on a CFX96 Touch Real-Time PCR Detection System (Bio-Rad Laboratories, Hercules, CA, USA). PCR reactions were prepared in a final volume of 20 μL using 2× SsoAdvanced™ Universal SYBR^®^ Green Supermix (2×) and PrimePCR™ SYBR Green Assays as follows: PrimePCR™ Template for SYBR^®^ Green Assay: *XYLT1*, Human (qHsaCIP0030871); PrimePCR™ Template for SYBR^®^ Green Assay: *GAPDH*, Human (qHsaCEP0041396). Thermal cycling conditions were 95 °C for 30 s, 40 cycles of denaturation at 95 °C for 30 s, and annealing and extension at 60 °C for 30 s. Each measurement was performed in triplicate and the threshold cycle (Ct) the fractional cycle number at which the amount of amplified target reached a fixed threshold was determined. The relative expression of *XYLT1* was calculated using the Livak method (2^−ΔΔCt^ method), with *GAPDH* serving as the internal control for normalization.

### 4.4. Western Blot Analyses

For Western blot analysis, patient-derived fibroblasts grown in 75 cm^2^ flasks and transfected with 100 nM of the best-performing gapmers were harvested 72 and 96 h after transfection. Total protein extracts were homogenized in a RIPA buffer (25 mM Tris, pH = 7.6, 150 mM NaCl, 1% nonidet P40, 1% sodium deoxycholate and 0.1% SDS) supplemented with a 0.01% Protease Inhibitor Cocktail (Sigma-Aldrich^®^, St. Louis, MO, USA). Cell lysis was achieved by keeping the solution on ice for 1 h with resuspension every 10 min, followed by brief centrifugation. Total protein quantification was carried out with the Pierce^TM^ BCA Protein Assay Kit (Thermo Scientific^TM^, Waltham, MA, USA). A total of 20 µg of protein was loaded on a 4–15% Mini-PROTEAN^®^ TGX Stain-Free™ Protein Gels (Bio-Rad Laboratories, Hercules, CA, USA). After electrophoresis, proteins were transferred to a nitrocellulose membrane using a Trans-Blot Turbo Transfer System (Bio-Rad Laboratories, Hercules, CA, USA), for 30 min at 20 mA. The membrane was then incubated with blocking solution (5% NFDM/TBS-T) for 2 h at RT, with gentle agitation on a shaker. The membrane was cut into two sections, around the 70 kDa molecular weight marker, and incubated overnight at 4 °C on a shaker with the primary antibodies rabbit anti-*XYLT1* (1:1000, Catalogue# 043941, US Biological, Salem, MA, USA) and mouse anti-α-tubulin (1:5000, Catalogue# T6199, Sigma-Aldrich, St. Louis, MO, USA), with the latter as a control. The next day, after washing in 0.5% TBS-T, bound antibodies were detected using HRP-conjugated goat anti-mouse IgG (1:5000, Catalogue# 31431, Invitrogen™ Thermo Fisher Scientific, Waltham, MA, USA) and mouse anti-rabbit IgG (1:5000, Catalogue# sc-2357, Santa Cruz Biotechnology Dallas, TX, USA) for 1 h at RT. Membranes were incubated with ECL solution, and an image was captured using the ChemiDoc^TM^ XRS+ imaging system (Bio-Rad Laboratories, Hercules, CA, USA).

### 4.5. Heparan Sulfate Quantification

HS and DS levels were quantified by liquid chromatography–tandem mass spectrometry (LC-MS/MS) in MPS IIIC cell homogenates, transfected with the best-performing gapmer ASOs, after the butanolysis reaction, according to the method originally described by Forni and co-workers for urine samples [49] and slightly modified in house to accommodate for cell lysates [50]. Briefly, cell homogenates were prepared by sonication, and their protein concentration was determined using the same method described for the Western blot analyses. Each cell homogenate was divided into two different plates, one for HS and another for DS. Samples were then dried under a stream of nitrogen, and 75 µL of 3 N HCl in N-butanol was added to each plate well.

For HS measurements, samples were incubated for 60 min at 90 °C, while for DS measurements, samples were heated for 25 min at 65 °C. After those incubations, samples were cooled back to RT for 10 min and dried again using the same method. Then, 30:70 water/acetonitrile (*v*/*v*) solution was added to samples: 100 µL for HS and 250 µL for DS quantification. After a brief vortex, the HS samples were combined with their respective DS counterparts and vortexed again. Finally, dimers derived from butanolysis reactions were separated by HPLC using a gradient of acetonitrile and water (LC column: Gemini^®^ 3 µm C6-Phenyl 110 Å, 100 × 2 mm, from Phenomenex, Torrance, CA, USA) and detected using a triple quadrupole mass spectrometer, API4000 QTRAP (Sciex, Danaher Corporation, Washington, DC, USA). Sample quantification was performed by interpolation from a calibration curve (covering a concentration range from 0.39 to 50 µg/mL).

### 4.6. Statistical Analyses

All experiments were performed at least three times, with data presented as mean ± standard deviation. To compare the means between groups, one-way ANOVA was performed, followed by Tukey’s multiple comparisons test. Differences were considered statistically significant if the *p*-value was less than 0.05. All tests were performed using GraphPad Prism^®^ software for Windows (version 8.0.1).

## 5. Conclusions

This study provides a proof of concept for the use of gapmer ASOs in reducing HS accumulation in MPS IIIC fibroblasts, supporting the further preclinical development of gSRT for MPS IIIC and potentially other MPS III subtypes. By targeting the biosynthetic pathway of all GAGs, this approach not only addresses the primary cause of cellular damage in MPS IIIC that is caused by HS accumulation but also opens up avenues for a broader therapeutic strategy within the field of lysosomal storage disorders. The results presented here underline the potential of ASOs to fulfill unmet clinical needs for CNS-targeted therapies, providing a foundation for future investigations into ASO-based interventions in MPS III.

Taken together, our results indicate that this approach is able to promote a reduction in mRNA levels of our target gene and, consequently, of our target protein, which translate into a notable reduction in the HS synthetic pathway after 6 and 10 days. Although further research is needed, this is a promising approach with the potential to be used as a complementary therapy to obtain synergic effects with other treatments developed to accelerate the rate of HS degradation and/or excretion out of the cell. Furthermore, in the future, our strategy could be a viable option not only for older patients (in helping to slow disease progression), but also for younger patients awaiting more permanent solutions, such as gene therapy or gene editing.

## Figures and Tables

**Table 1:** Sequences of gapmer ASOs and their target exon in the *XYLT1* gene. NA—not applicable.

Gapmer Designation	Gapmer Sequence (5′ -> 3′)	Target Exon
Scramble	CCTATAGGACTATCCAGGAA	NA
G1	CGATGCAGGTAATTAGAGCG	5
G2	GTCAGCAAGGAAGTAGAGGT	10
G3	GTCAGGCTCATCGTAGACAT	9
G4	GCAACACATCTGGCTAGGAT	12
G5	ACAATCTTGGGACCAGGAGA	3′UTR

## Data Availability

Data are contained within the article and Supplementary Materials.

## Supplementary Materials

The following supporting information can be downloaded at: https://www.mdpi.com/article/10.3390/ijms26031273/s1.

## Abbreviations

The following abbreviations are used in this manuscript:

LSDs	Lysosomal storage disorders
MPS III	Mucopolysaccharidosis type III
gSRT	Genetic substrate reduction therapy
ASOs	Antisense oligonucleotides
HS	Heparan sulfate
XT-I	Xylosyltransferase I
XT-II	Xylosyltransferase II
PS	Phosphorothioate
IT	Intrathecal
MPSs	Mucopolysaccharidoses
GAGs	Glycosaminoglycans
ERTs	Enzyme replacement therapies
BBB	Blood–brain barrier
SRTs	Substrate reduction therapies
CSF	Cerebrospinal fluid
DMEM	Dulbecco’s Modified Eagle Medium
FBS	Fetal bovine serum
LC-MS/MS	Liquid chromatography-tandem mass spectrometry
NT	Non-transfected
HDFa	Adult Human Dermal Fibroblasts
DBSD	Desbuquois skeletal dysplasia
RT	Room temperature
